# Association between three‐dimensional gait kinematics and joint‐line inclination in osteoarthritic knees compared with normal knees: An epidemiological study

**DOI:** 10.1002/jeo2.12040

**Published:** 2024-06-11

**Authors:** Fangzhou Chi, Tomoharu Mochizuki, Hiroshi Koga, Go Omori, Katsutoshi Nishino, Shigeru Takagi, Yoshio Koga, Hiroyuki Kawashima

**Affiliations:** ^1^ Division of Orthopedic Surgery, Graduate School of Medical and Dental Sciences Niigata University Niigata Japan; ^2^ Department of Orthopedic Surgery, The First Affiliated Hospital Harbin Medical University Harbin China; ^3^ Division of Musculoskeletal Science for Frailty, Graduate School of Medical and Dental Sciences Niigata University Niigata Japan; ^4^ Department of Health and Sports Niigata University of Health and Welfare Niigata Japan; ^5^ Niigata Institute for Health and Sports Medicine Niigata Japan; ^6^ Niohji Onsen Clinic Niigata Japan

**Keywords:** epidemiological study, gait analysis, joint‐line inclination, KL grade, knee osteoarthritis

## Abstract

**Purpose:**

No report has proven how tibial and femoral joint‐line inclinations affect thigh and shank motion, respectively, according to Kellgren–Lawrence grade in motion analysis with a sufficient sample size. Therefore, this study aimed to evaluate the motion of the thigh and shank individually from the ground and the relative motion between bones in a large‐sample motion analysis to determine the differences between normal and osteoarthritic knees and examine the effects of tibial and femoral joint‐line inclination on motion according to osteoarthritis (OA) grade.

**Methods:**

Of 459 participants with healthy knees and varus knee OA undergoing three‐dimensional gait analysis, 383 (218 females and 165 males) with an average age of 68 ± 13 years were selected. Gait analysis was performed using a motion–capture system. The six degrees of freedom motion parameters of the knee in the Grood and world coordinate systems and the joint‐line inclination in the standing radiographs were measured.

**Results:**

Osteoarthritic knees demonstrated a relative motion different from that of normal knees, with responsibility for the thigh in the sagittal and rotational planes and the thigh and shank in the coronal plane. The involvement of joint‐line inclination in motion was mainly on the tibial side, and the effect was minimal in normal knees.

**Conclusions:**

The details of the relative motion of both the thigh and shank can be clarified by analysing individual motions to determine the responsible part. The tibial joint‐line affected knee motion: however, the effect was minimal in normal knees. This finding implies that if physical ability can be improved, the negative effects of deformity in osteoarthritic knees may be compensated for.

**Level of Evidence:**

Level Ⅱ.

**Abbreviations**
3D: three dimensional
ANOVA: analysis of variance
BMI: body mass index
FTA: femorotibial angle
JCLA: joint‐line convergence angle
KL: Kellgren–Lawrence
OA: osteoarthritis
TPA: tibial plateau angle

## INTRODUCTION

The diversity between joint‐line inclination and lower extremity alignment in knee osteoarthritis (OA) has received much attention in recent years [3, 5, 6, 7]. This diversity indicates that the pathophysiology of knee OA is not uniform. In practice, not everyone will develop knee OA even if the joint‐line inclination in a knee joint is steep. If muscular strength and other physical functions are used to compensate for the slope of the joint line, the burden on the knee joint may be reduced and knee OA may not develop or progress.

Even with steep joint‐line inclination in a knee joint, it is assumed that the effect of age and muscle strength on movement will vary. As OA grade progresses over time, it is inevitably, greatly affected by age, causing muscle strength deterioration [2, 16]. Younger patients may control motion with high muscle power and physical ability, even if the tibial joint‐line inclination is steep; however, in older patients with reduced physical ability, the effect of structural failure is expected to impact movement significantly. Therefore, the effect of joint‐line inclination on motion is highly likely to differ according to each Kellgren–Lawrence (KL) grade [8] proportional to age. To date, no report has proven how tibial and femoral joint‐line inclinations affect thigh and shank motion, respectively, according to KL grade in motion analysis with a sufficient sample size.

An epidemiological study on knee OA was conducted between 1979 and 2022 using the Matsudai Knee Osteoarthritis Survey [4, 20]. The study provided time‐course changes and revealed several causes of knee OA onset and progression [4, 20]. In this survey, three‐dimensional (3D) motion analysis has been recently performed using a motion capture system to investigate the association between motion and other factors with the onset and progression of OA.

We hypothesised that the kinematics in normal and OA knees would differ, and the effects of tibial and femoral joint‐line inclinations on motion would also differ according to the KL grade. This study aimed to evaluate the motion of the thigh and shank individually from the ground and the relative motion between bones in motion analysis with a large sample size to (1) determine the differences in motion between normal and OA knees and (2) examine the effects of tibial and femoral joint‐line inclinations on motion according to the KL grade.

## MATERIALS AND METHODS

The Institutional Review Board of our University approved this study. All the participants provided informed consent before participating in the survey.

The Matsudai Knee Osteoarthritis Survey is a population‐based epidemiological survey conducted in 1979. The first five surveys were conducted at 7‐year intervals (1979, 1986, 1993, 2000 and 2007). The sixth to tenth surveys were conducted at 3‐year intervals (2010, 2013, 2016, 2019 and 2022). The present study analysed the gait data from the 2019 survey.

Of 459 participants with healthy knees and varus knee OA who underwent 3D gait analysis in the 2019 survey, 383 with no missing data were selected. The exclusion criteria were lateral knee OA, high tibial osteotomy or knee arthroplasty. This study included 218 females (average age of 67 years [range: 23–94 years] and body mass index [BMI] of 23.1 kg/m^2^ [range: 15.6–39.0 kg/m^2^]) and 165 males (average age of 70 years [range: 21–92 years] and BMI of 23.5 kg/m^2^ [range: 17.8–36.3 kg/m^2^]).

The anteroposterior radiographs of the knee joints were obtained in the standing position with maximum extension. Radiographic OA was classified as grades 0–4 using the KL classification [8]. In the present study, patients with knee OA were divided into three groups: ‘non‐OA’ (grades 0 and 1), ‘early OA’ (grade 2) and ‘advanced OA’ (grades 3 and 4). Two trained orthopaedic surgeons graded the radiographs separately. Disagreements were discussed and adjudicated based on examiners' consensus. The weighted *κ* coefficient was 0.79 for knees classified as KL grade ≤2 and 0.98 for those classified as KL grades 3 and 4. This study revealed grades 0 and 1 in 109 (28.5%) and 108 (28.2%) knees, grade 2 in 64 (16.7%) and 44 (11.5%) knees and grades 3 and 4 in 45 (11.7%) and 13 (3.4%) knees of female and male participants, respectively.

**Figure 1:**
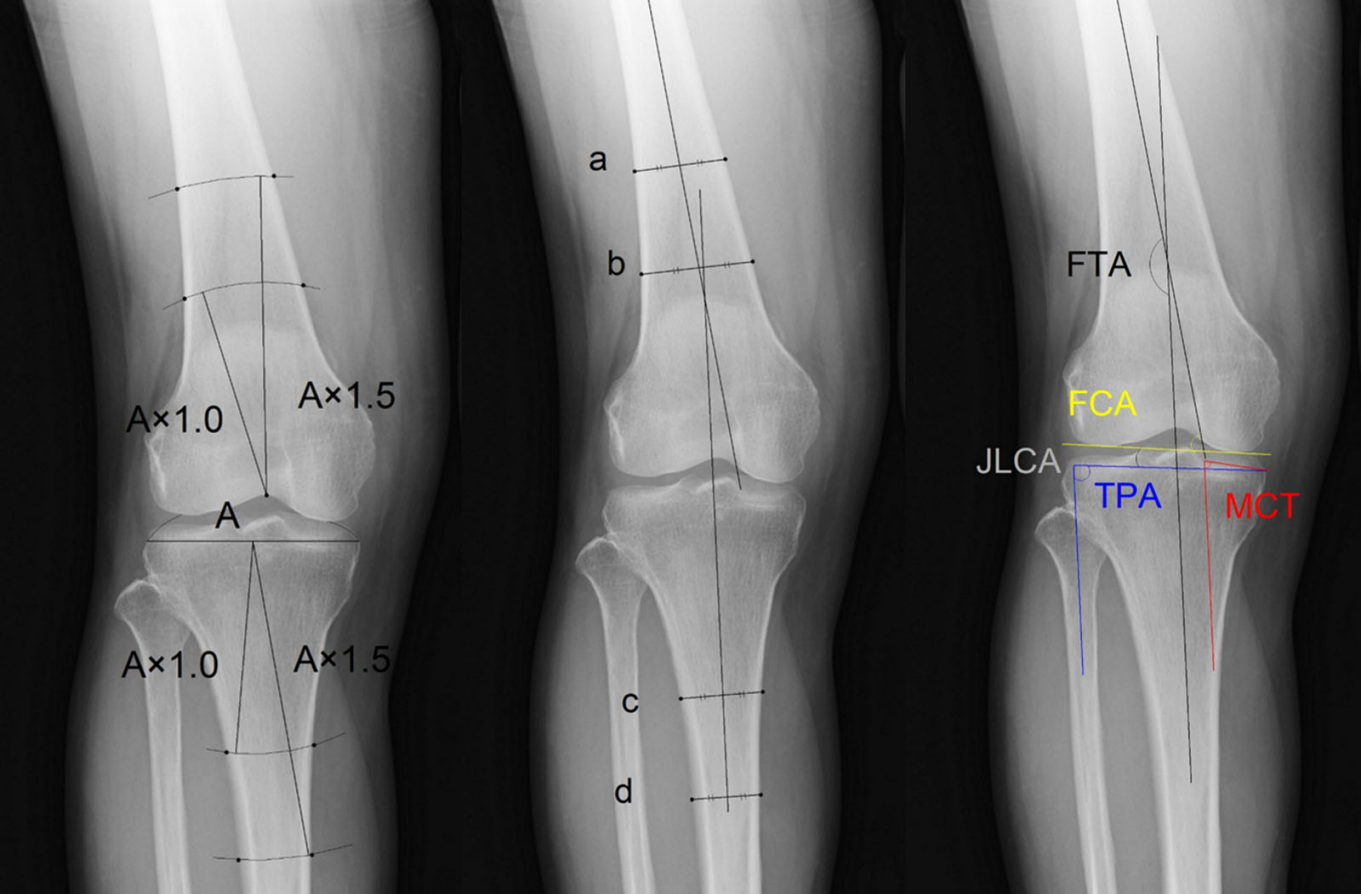
Static parameters.

The anatomical axes of the femur and tibia were determined as previously described [4] (Figure 1). The distance between the medial and lateral edges of the tibial plateau was defined as the width of the tibial plateau (line A). On the tibial side, circles with radii of 1.0 and 1.5 times A were drawn from the midpoint of line A, and the intersection points of the most lateral and medial cortical bone of the tibia with the circles were marked. On the femoral side, circles with radii of 1.0 and 1.5 times A were drawn from the intercondylar area of the femur, and the intersection points of the outermost and innermost cortical bone of the femur with the circles were marked. The points marked on the same circle were connected to form lines referred to as a, b, c and d. The line passing through the midpoints of lines a and b was defined as the anatomical axis of the femur, whereas that passing through the midpoints of lines c and d was defined as the anatomical axis of the tibia. The femorotibial angle (FTA) was defined as the angle formed by both anatomical axes. A larger FTA indicated a larger varus angle. The tibial plateau angle (TPA) was defined as the angle formed by the tibial anatomical axis and articular surface. The medial compartment of the TPA was defined as the angle formed by the line connecting the medial end of the proximal articular surface of the tibia and that of the intercondylar eminence and the tibial anatomical axis. The femoral condylar angle was defined as the angle formed by the femoral anatomical axis and articular surface. The joint‐line convergence angle (JLCA) was defined as the angle formed by the femoral and tibial coronal joint surfaces. The JLCA was set to a positive value when the intersection of the femoral and tibial joint surfaces was on the medial side of the knee joint and to a negative value when the intersection was on the lateral side (Figure 1).

**Figure 2:**
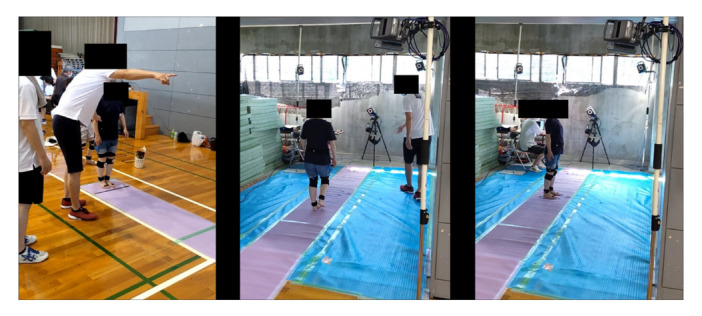
Motion analysis in the epidemiological study.

The basic methodology of the present study has been previously described [15] (Figure 2). A total of 18 skin markers were attached to each participant. An experienced investigator attached thigh markers to the medial and lateral femoral epicondyles and anterior superior iliac spine. Shank markers were placed on the medial and lateral malleoli, the fibular head and the tibial tuberosity. The participants walked along an 8.0 m flat lane at a preferred speed. Table 1 presents the stride time (duration of one gait cycle), stride length and stride length ratio standardised by body height. The world coordinate system of the motion capture system (VICON612; Vicon Motion Systems Ltd.) was set at the centre of the lane, where the participants reached a constant walking speed. The gait was captured at a sampling rate of 250 Hz. The 3D positions of the markers were determined as previously described [15]. The positions were smoothed using a fourth‐order zero‐lag Butterworth filter (6 Hz) [21]. Regarding accuracy, the detection error of the 3D position of the marker was <0.7 mm under static conditions of a capture space of 3.0 m × 4.0 m × 2.5 m with a retroreflective skin marker. The gait cycle was classified using five time points: initial contact, foot flat, heel‐rise, opposing initial contact and toe‐off. The stance phase was defined as the time from the initial contact to the toe‐off. In the stance phase, the time from the initial contact to foot flat was defined as the loading response phase (0%–20% of the stance phase), that from foot flat to heel‐rise was defined as the mid‐stance phase (20%–50% of the stance phase), that from heel‐rise to the opposing initial contact was defined as the terminal stance phase (50%–83% of the stance phase) and that from the opposing initial contact to the toe‐off was defined as the pressing phase (83%–100% of the stance phase) [17].

**Table 1:** Demographic data.

Variables	KL 0, 1 (*n* = 217)			KL 2 (*n* = 108)			KL 3, 4 (*n* = 58)			*p* Value
	Mean ± SD	95% CI		Mean ± SD	95% CI		Mean ± SD	95% CI		
Age (years)	64.1 ± 13.9	62.2	65.9	72.5 ± 8.0	71.0	74.1	77.4 ± 6.8	75.7	79.2	<0.001*
Body weight (kg)	56.7 ± 10.7	55.3	58.2	54.8 ± 10.1	52.8	56.7	55.8 ± 10.2	53.1	58.5	0.334
Body height (cm)	157.2 ± 8.8	156.0	158.4	153.5 ± 9.1	151.7	155.2	147.4 ± 8.0	145.3	149.5	<0.001*
Body mass index (kg/m^2^)	22.8 ± 3.0	22.4	23.2	23.1 ± 2.8	22.6	23.7	25.6 ± 3.8	24.6	26.6	<0.001*
Stride time (s)	1.1 ± 0.1	1.1	1.1	1.1 ± 0.1	1.0	1.1	1.1 ± 0.2	1.1	1.2	0.339
Stride length (m)	1.1 ± 0.2	1.1	1.1	1.1 ± 0.2	1.0	1.1	0.9 ± 0.2	0.9	1.0	<0.001*
Gait velocity (m/s)	1.0 ± 0.2	1.0	1.1	1.0 ± 0.2	1.0	1.1	0.9 ± 0.2	0.8	0.9	<0.001*
FTA (°)	177.7 ± 2.7	177.3	178.1	178.2 ± 3.1	177.6	178.8	181.6 ± 5.4	180.1	183.0	<0.001*
MCT (°)	76.9 ± 3.2	76.5	77.3	78.0 ± 3.4	77.4	78.7	75.1 ± 4.1	74.0	76.2	<0.001*
TPA (°)	83.3 ± 2.3	83.0	83.6	83.7 ± 2.5	83.2	84.2	81.6 ± 2.9	80.8	82.3	<0.001*
FCA (°)	80.2 ± 1.7	79.9	80.4	80.4 ± 1.9	80.0	80.8	80.5 ± 2.4	79.8	81.1	0.254
JLCA (°)	0.8 ± 1.7	0.6	1.1	1.1 ± 2.1	0.8	1.5	2.7 ± 2.6	2.0	3.3	<0.001*
Proportion (*n*)										
Sex (females:males)	109:108			64:44			45:13			0.001*

Abbreviations: 95% CI, 95% confidence interval; FCA, femoral condylar angle; FTA, femorotibial angle; JLCA, joint‐line convergence angle; KL, Kellgren–Lawrence classification; MCT, medial compartment of tibia plateau angle; *n*, subjects; Ratio of stride length, stride length/body height; SD, standard deviation; Stride time, time in one gait cycle; TPA, tibial plateau angle.

* *p* < 0.05.

**Figure 3:**
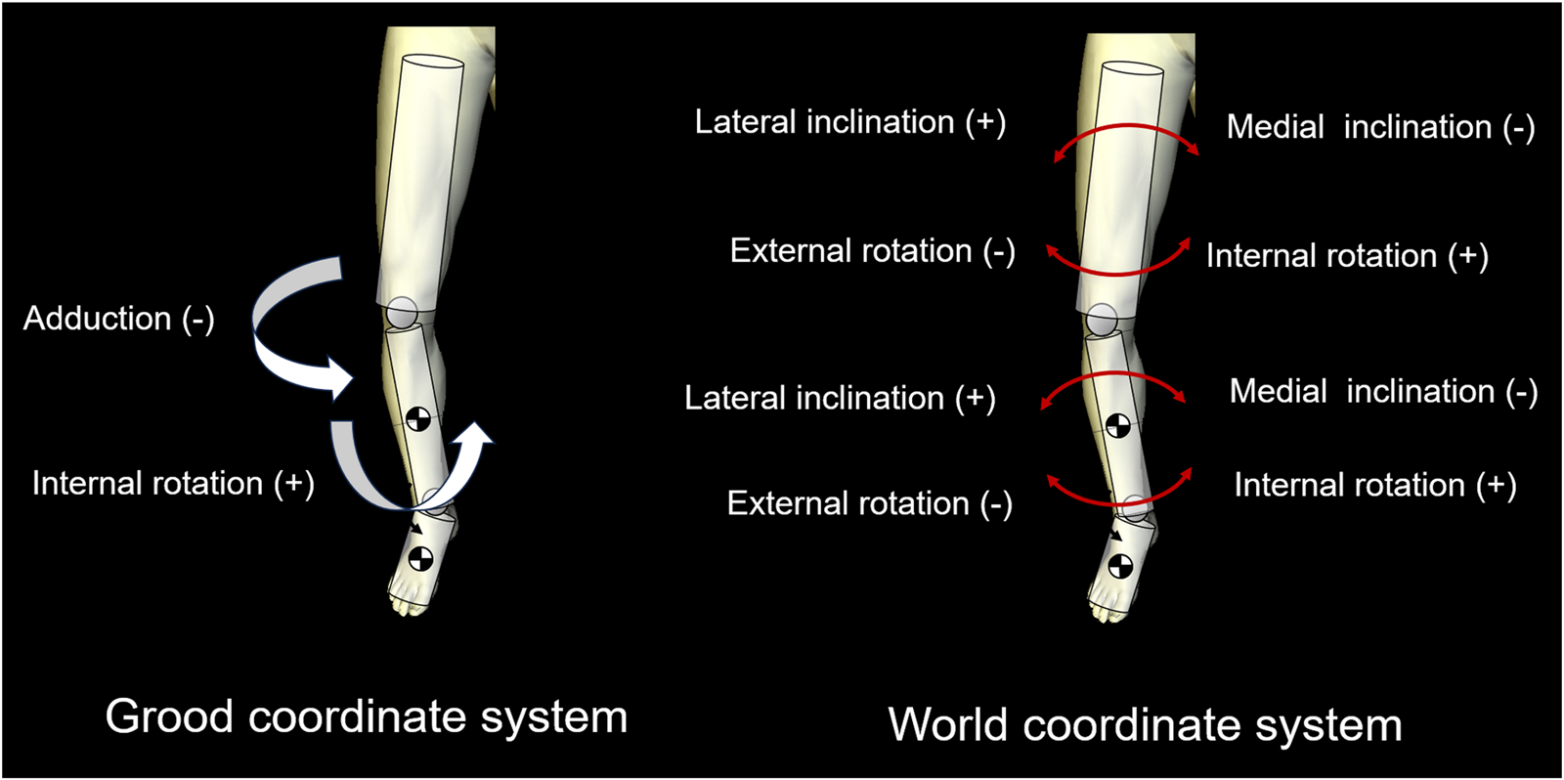
Kinematic parameters.

Regarding kinematic parameters (Figure 3), the shank motion relative to the thigh was calculated using the Grood coordinate system and the dynamic positions of the thigh and shank in the world coordinate system during the stance phase of the gait cycle were calculated. Each parameter was defined as positive in the Grood coordinate system when the shank relative to the thigh was in flexion, abduction or internal rotation. In the world coordinate system, each position relative to the ground was defined as the position of the thigh and shank relative to the *z, x* and *y* axes of the world coordinate system in the coronal (lateral inclination, +), sagittal (posterior inclination, +) and transverse (internal rotation, +) planes. The world coordinate system was described as follows: the *y* axis is the gait direction, the *z* axis is the gravity direction and the *x* axis is the cross‐product of the *y* and *z* axes. All the kinematics were evaluated as translations of the initial positions.

### Statistical analysis

**Figure 4:**
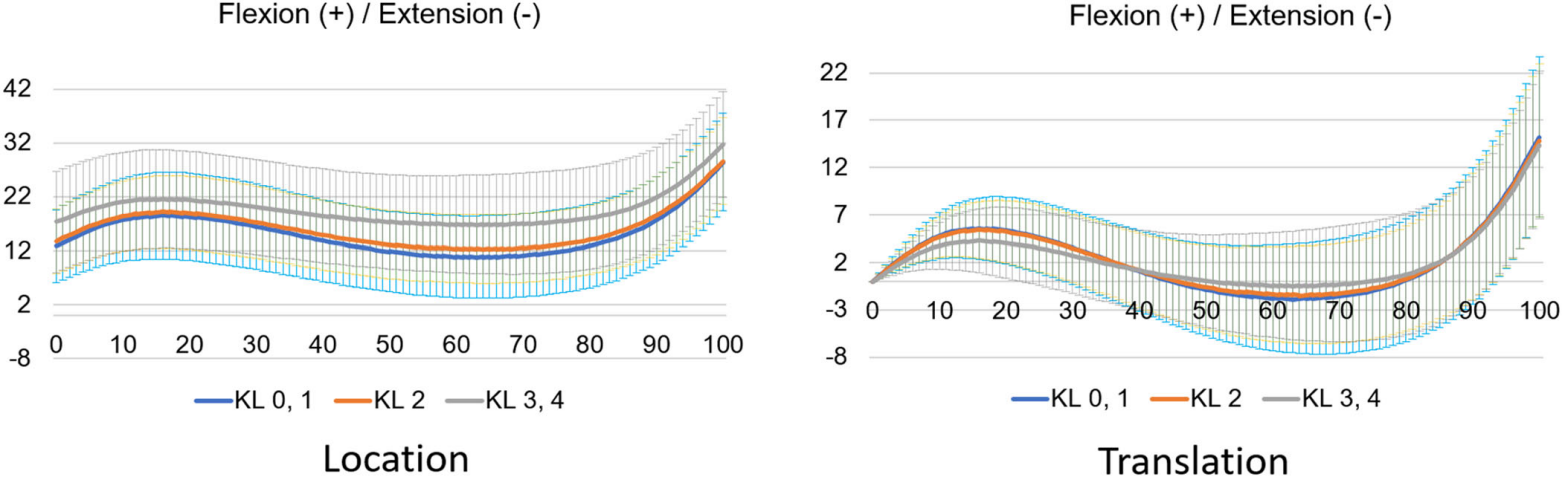
Kinematics of flexion–extension in the stance phase based on the Grood coordinate system.

**Figure 5:**
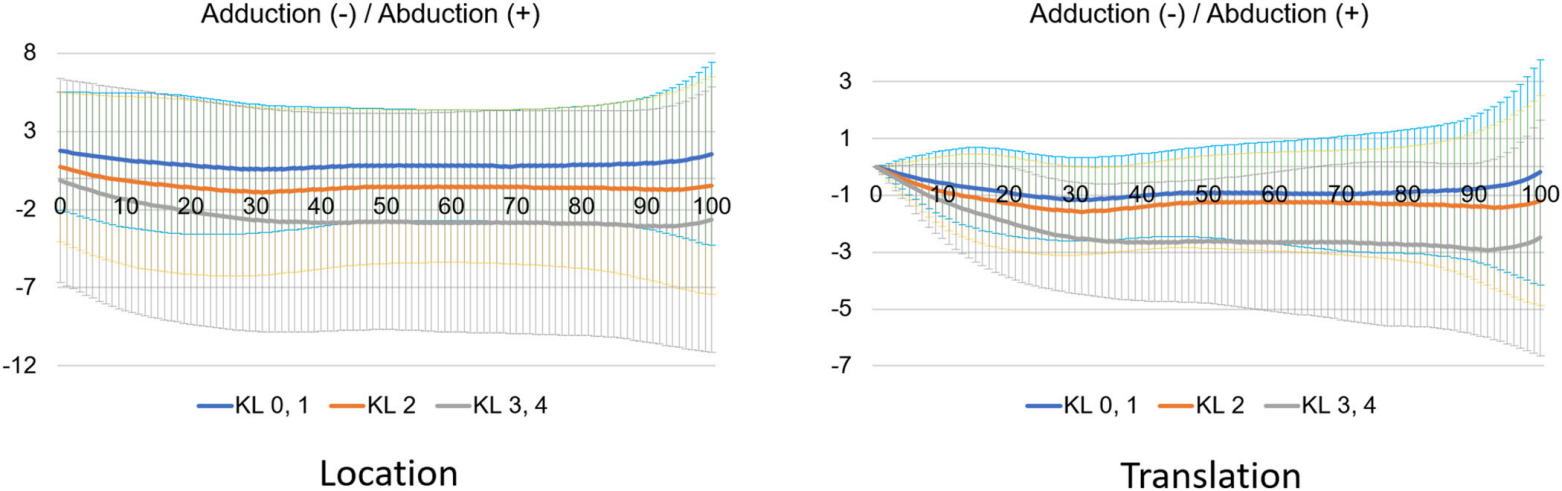
Kinematics of adduction–abduction in the stance phase based on the Grood coordinate system.

**Figure 6:**
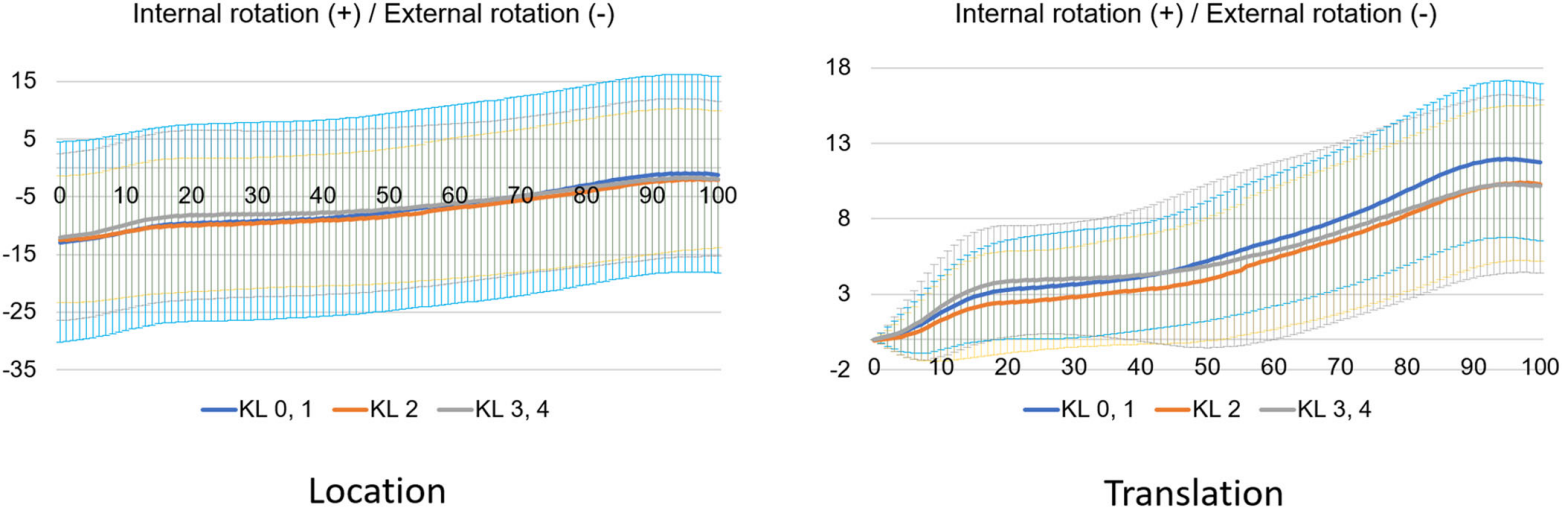
Kinematics of internal rotation–external rotation in the stance phase based on the Grood coordinate system.

**Figure 7:**
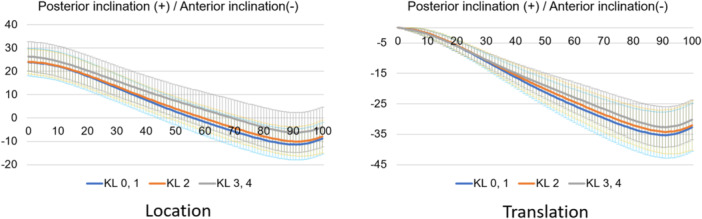
Femoral sagittal motion in the stance phase based on the world coordinate system.

**Figure 8:**
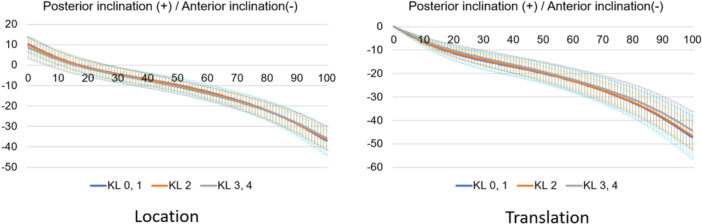
Tibial sagittal motion in the stance phase based on the world coordinate system.

**Figure 9:**
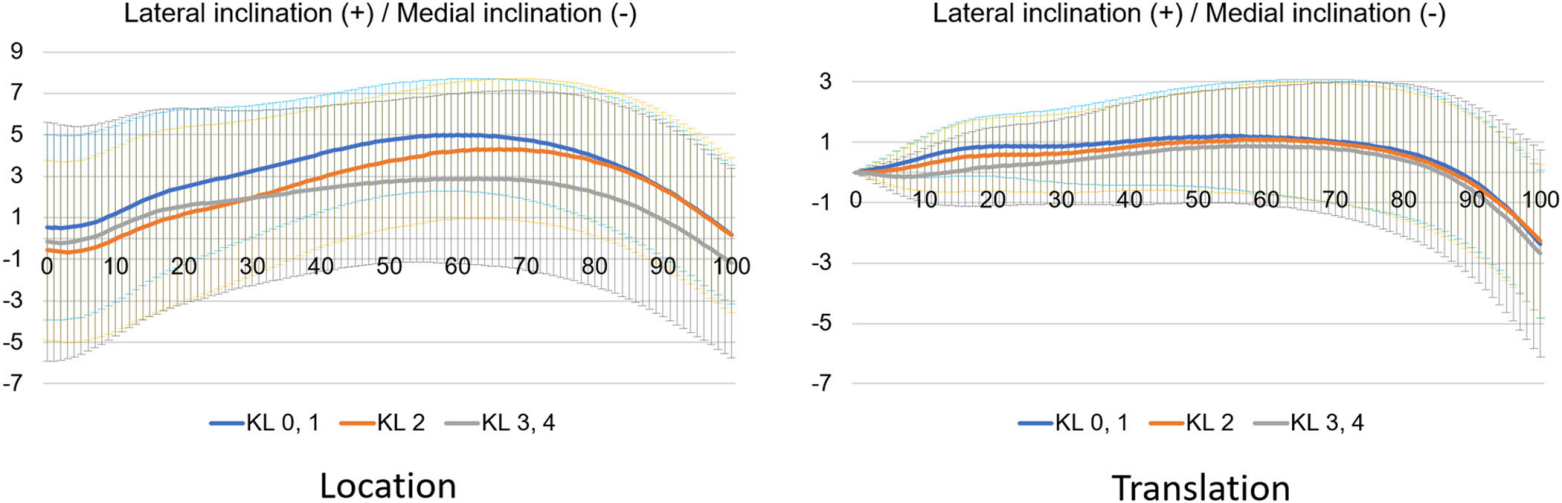
Femoral coronal motion in the stance phase based on the world coordinate system.

**Figure 10:**
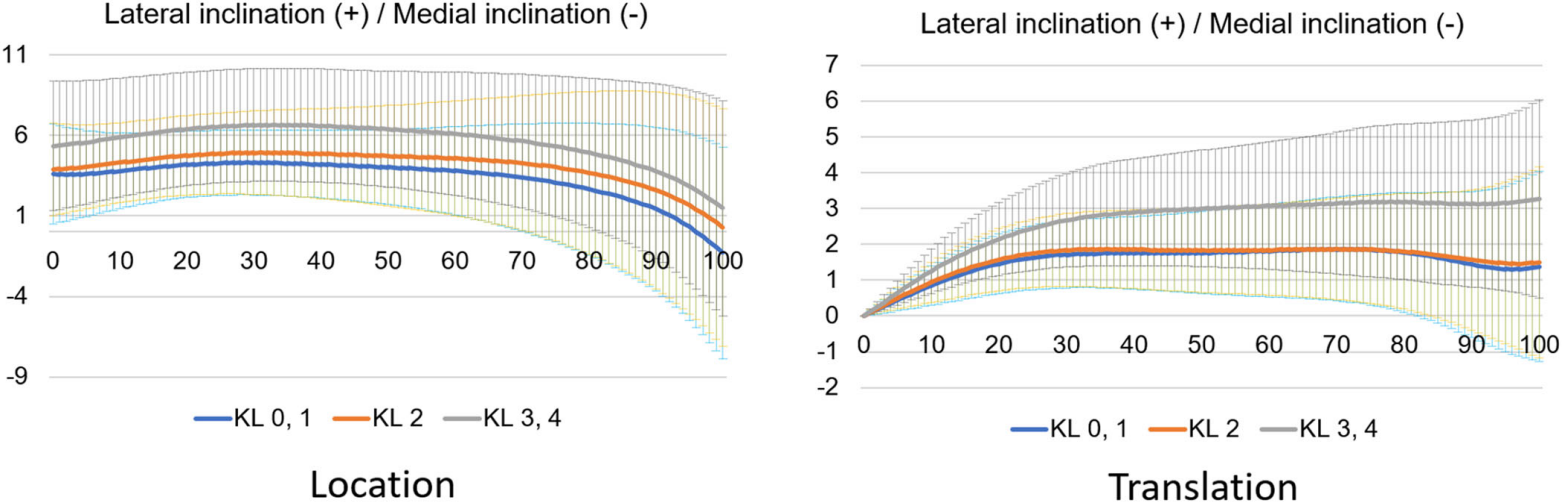
Tibial coronal motion in the stance phase based on the world coordinate system.

**Figure 11:**
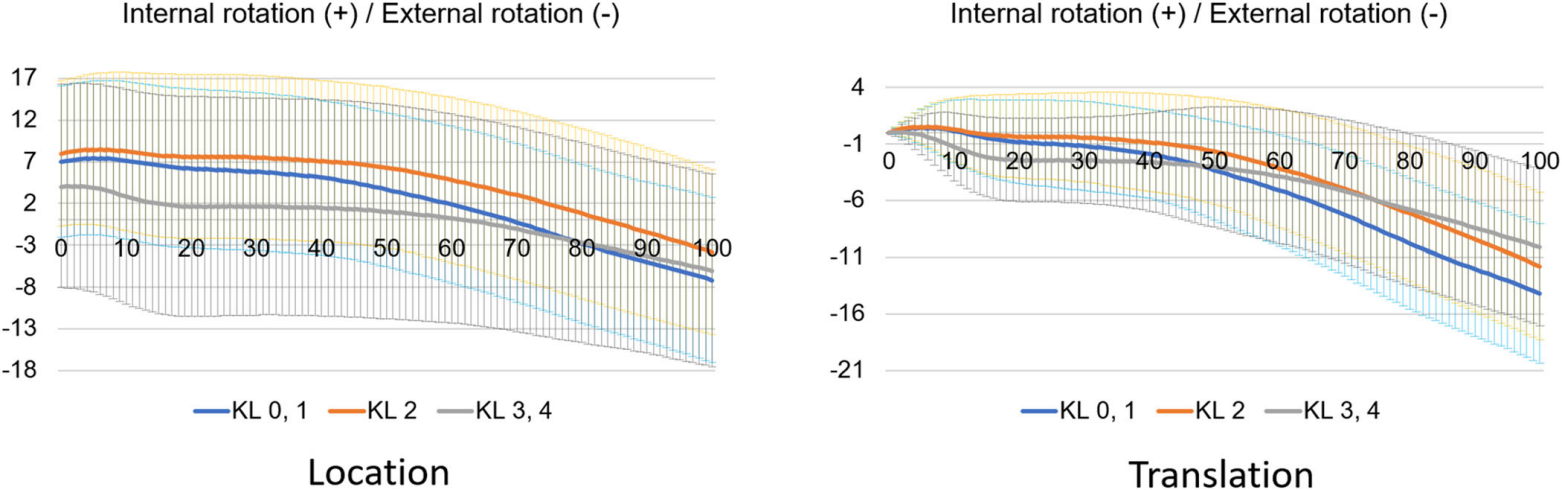
Femoral rotational motion in the stance phase based on the world coordinate system.

**Figure 12:**
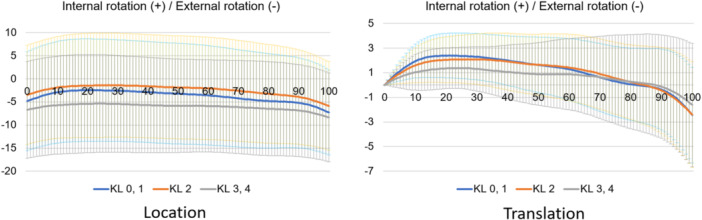
Tibial rotational motion in the stance phase based on the world coordinate system.

The Shapiro–Wilk test was used to examine the normality of the data. One‐way analysis of variance (ANOVA) with Tukey's post hoc test was performed on normally distributed data to compare differences amongst the three groups (Table 1). The Kruskal–Wallis test with the Bonferroni post hoc test was performed on non‐normally distributed data. Repeated‐measures ANOVA with Tukey's post hoc test was used to assess the differences in the overall kinematics (Tables 2 and 3; Figures 4, 5, 6, 7, 8, 9, 10, 11, 12). When the correlations between the static and motion parameters were analysed, Pearson's product–moment correlation coefficient was used for normally distributed data, whereas Spearman's rank correlation coefficient was used for non‐normally distributed data (Tables 4, 5, 6). Statistical significance was set at *p* < 0.05. Statistical Package for the Social Sciences software (version 27; SPSS Inc.) was used for statistical analyses.

**Table 2:** Comparison of overall kinematics in location using repeated measures ANOVA with a post hoc test.

Variables	*p* Value			
	All groups	KL 0, 1 vs. KL 2	KL 0, 1 vs. KL 3, 4	KL 2 vs. KL 3, 4
Grood coordinate system, loading response phase				
Flexion–extension	0.006*	0.679	0.004*	0.057
Adduction–abduction	0.002*	0.076	0.002*	0.303
Internal–external rotation	0.867	1.000	0.862	0.888
Grood coordinate system, stance phase				
Flexion–extension	<0.001*	0.433	<0.001*	0.005*
Adduction–abduction	<0.001*	0.033*	<0.001*	0.034*
Internal rotation–external rotation	0.909	0.945	0.978	0.909
World coordinate system, loading response phase				
Posterior–anterior inclination, femur	0.036*	0.938	0.029*	0.095
Posterior–anterior inclination, tibia	0.158	0.973	0.139	0.262
Lateral–medial inclination, femur	0.050	0.044*	0.487	0.765
Lateral–medial inclination, tibia	<0.001*	0.269	<0.001*	0.001*
Internal–external rotation, femur	0.005*	0.589	0.016*	0.004*
Internal–external rotation, tibia	0.110	0.670	0.234	0.092
World coordinate system, stance phase				
Posterior–anterior inclination, femur	<0.001*	0.283	<0.001*	0.002*
Posterior–anterior inclination, tibia	0.499	0.991	0.475	0.600
Lateral–medial inclination, femur	0.001*	0.047*	0.002*	0.353
Lateral–medial inclination, tibia	<0.001*	0.057	<0.001*	0.004*
Internal–external rotation, femur	0.009*	0.084	0.258	0.009*
Internal–external rotation, tibia	0.107	0.542	0.296	0.088

Abbreviations: ANOVA, analysis of variance; KL, Kellgren–Lawrence classification.

* *p* < 0.05.

**Table 3:** Comparison of overall kinematics in translation using repeated measures ANOVA with a post hoc test.

Variables	*p* Value			
	All groups	KL 0, 1 vs. KL 2	KL 0, 1 vs. KL 3, 4	KL 2 vs. KL 3, 4
Grood coordinate system, loading response phase				
Flexion–extension	0.013*	0.808	0.009*	0.065
Adduction–abduction	<0.001*	0.102	<0.001*	0.109
Internal–external rotation	0.050	0.140	0.576	0.059
Grood coordinate system, stance phase				
Flexion–extension	0.971	0.971	0.991	0.999
Adduction–abduction	<0.001*	0.079	<0.001*	<0.001*
Internal rotation–external rotation	0.023*	0.017*	0.688	0.428
World coordinate system, loading response phase				
Posterior–anterior inclination, femur	0.382	0.990	0.397	0.410
Posterior–anterior inclination, tibia	0.009*	0.638	0.006*	0.084
Lateral–medial inclination, femur	<0.001*	0.027*	<0.001*	0.020*
Lateral–medial inclination, tibia	<0.001*	0.376	<0.001*	0.001*
Internal–external rotation, femur	0.001*	0.754	0.003*	0.001*
Internal–external rotation, tibia	<0.001*	0.128	<0.001*	0.035*
World coordinate system, stance phase				
Posterior–anterior inclination, femur	0.014*	0.224	0.015*	0.375
Posterior–anterior inclination, tibia	0.166	0.899	0.141	0.339
Lateral–medial inclination, femur	0.149	0.637	0.137	0.526
Lateral–medial inclination, tibia	<0.001*	0.842	<0.001*	<0.001*
Internal–external rotation, femur	0.004*	0.003*	0.427	0.409
Internal–external rotation, tibia	0.332	0.976	0.304	0.461

Abbreviations: ANOVA, analysis of variance; KL, Kellgren–Lawrence classification.

* *p* < 0.05.

**Table 4:** Correlation between static and kinematic parameters in location.

Parameters (average data in kinematics)	FTA		MCT		TPA		FCA		JLCA	
	CC	*p*	CC	*p*	CC	*p*	CC	*p*	CC	*p*
Grood coordinate system, loading response phase										
Flexion–extension	−0.008	0.883	0.030	0.555	−0.011	0.833	−0.039	0.445	0.057	0.267
Adduction–abduction	−0.253	<0.001*	0.213	<0.001*	0.247	<0.001*	−0.066	0.200	−0.031	0.539
Internal–external rotation	−0.032	0.529	−0.097	0.059	−0.069	0.177	−0.083	0.104	−0.020	0.699
Grood coordinate system, stance phase										
Flexion–extension	0.024	0.639	0.015	0.764	−0.050	0.334	−0.046	0.371	0.084	0.099
Adduction–abduction	−0.302	<0.001*	0.229	<0.001*	0.273	<0.001*	−0.089	0.083	−0.077	0.132
Internal rotation–external rotation	−0.020	0.698	−0.106	0.037*	−0.079	0.122	−0.070	0.169	−0.020	0.700
World coordinate system, loading response phase										
Posterior–anterior inclination, femur	−0.044	0.390	0.044	0.391	−0.002	0.971	−0.066	0.200	0.041	0.428
Posterior–anterior inclination, tibia	−0.023	0.654	−0.018	0.719	0.014	0.790	−0.029	0.574	0.002	0.962
Lateral–medial inclination, femur	−0.176	0.001*	0.027	0.600	0.036	0.479	−0.151	0.003*	−0.082	0.107
Lateral–medial inclination, tibia	0.296	<0.001*	−0.176	0.001*	−0.268	<0.001*	0.095	0.062	0.096	0.062
Internal–external rotation, femur	−0.166	0.001*	0.161	0.002*	0.200	<0.001*	−0.012	0.820	−0.053	0.299
Internal–external rotation, tibia	−0.071	0.167	−0.023	0.654	0.022	0.671	−0.064	0.209	−0.048	0.346
World coordinate system, stance phase										
Posterior–anterior inclination, femur	0.010	0.842	−0.003	0.957	−0.064	0.211	−0.040	0.439	0.059	0.247
Posterior–anterior inclination, tibia	−0.030	0.562	−0.080	0.117	−0.036	0.483	−0.013	0.794	−0.106	0.038*
Lateral–medial inclination, femur	−0.312	<0.001*	0.147	0.004*	0.196	<0.001*	−0.164	0.001*	−0.076	0.137
Lateral–medial inclination, tibia	0.205	<0.001*	−0.191	<0.001*	−0.235	<0.001*	0.056	0.273	0.063	0.217
Internal–external rotation, femur	−0.130	0.011*	0.147	0.004*	0.180	<0.001*	0.000	0.993	−0.038	0.453
Internal–external rotation, tibia	−0.070	0.169	−0.022	0.670	0.023	0.655	−0.061	0.235	−0.047	0.359

Abbreviations: CC, correlation coefficient; FCA, femoral condylar angle; FTA, femorotibial angle; JLCA, joint‐line convergence angle; MCT, medial compartment of tibia plateau angle; TPA, tibial plateau angle.

* *p* < 0.05.

**Table 5:** Correlation between static and kinematic parameters in translation.

Parameters (average data)	FTA		MCT		TPA		FCA		JLCA	
	CC	*p*	CC	*p*	CC	*p*	CC	*p*	CC	*p*
Grood coordinate system, loading response phase										
Flexion–extension	−0.047	0.355	0.106	0.039*	0.108	0.035*	−0.029	0.567	0.034	0.513
Adduction–abduction	−0.092	0.074	0.144	0.005*	0.161	0.002*	−0.024	0.636	0.006	0.901
Internal–external rotation	0.027	0.596	−0.065	0.207	−0.119	0.020*	0.003	0.946	−0.107	0.036*
Grood coordinate system, stance phase										
Flexion–extension	0.080	0.116	0.012	0.821	−0.025	0.629	0.009	0.858	0.113	0.027*
Adduction–abduction	−0.147	0.004*	0.113	0.028*	0.130	0.011*	−0.054	0.291	−0.129	0.011*
Internal rotation–external rotation	0.080	0.118	−0.099	0.054	−0.132	0.010*	0.046	0.371	−0.050	0.333
World coordinate system, loading response phase										
Posterior–anterior inclination, femur	−0.005	0.927	0.082	0.107	0.054	0.294	−0.006	0.914	0.030	0.561
Posterior–anterior inclination, tibia	0.071	0.168	−0.087	0.088	−0.115	0.024*	0.057	0.266	−0.032	0.538
Lateral–medial inclination, femur	−0.044	0.393	0.092	0.072	0.122	0.017*	−0.007	0.896	0.051	0.319
Lateral–medial inclination, tibia	0.077	0.134	−0.074	0.150	−0.114	0.025*	0.039	0.446	0.008	0.870
Internal–external rotation, femur	−0.026	0.617	0.098	0.056	0.140	0.006*	−0.028	0.586	0.110	0.031*
Internal–external rotation, tibia	−0.014	0.785	0.043	0.402	0.032	0.529	−0.048	0.353	0.027	0.593
World coordinate system, stance phase										
Posterior–anterior inclination, femur	0.098	0.057	−0.045	0.381	−0.094	0.066	0.050	0.330	0.039	0.449
Posterior–anterior inclination, tibia	0.022	0.672	−0.064	0.209	−0.064	0.214	0.068	0.184	−0.085	0.096
Lateral–medial inclination, femur	−0.014	0.791	0.022	0.662	0.011	0.825	0.002	0.964	0.016	0.748
Lateral–medial inclination, tibia	0.141	0.006*	−0.077	0.134	−0.115	0.025*	0.089	0.080	0.115	0.025*
Internal–external rotation, Femur	0.057	0.264	0.008	0.873	0.008	0.881	0.001	0.977	0.093	0.070
Internal–external rotation, Tibia	0.008	0.869	0.032	0.531	0.013	0.794	−0.002	0.967	0.049	0.336

Abbreviations: CC, correlation coefficient; FCA, femoral condylar angle; FTA, femorotibial angle; JLCA, joint‐line convergence angle; MCT, medial compartment of tibia plateau angle; TPA, tibial plateau angle.

* *p* < 0.05.

**Table 6:** Correlation between static and kinematic parameters in the coronal plane for each KL grade.

	KL 0, 1 (*n* = 217)				KL 2 (*n* = 108)				KL 3, 4 (*n* = 58)			
Variables	CC	*p*	CC	*p*	CC	*p*	CC	*p*	CC	*p*	CC	*p*
	Loading		Stance		Loading		Stance		Loading		Stance	
Adduction–abduction in the Grood coordinate system, location (average data)												
FTA	−0.092	0.175	−0.125	0.066	−0.306	0.001*	−0.323	0.001*	−0.455	<0.001*	−0.495	<0.001*
MCT	0.129	0.058	0.122	0.074	0.282	0.003*	0.271	0.005*	0.584	<0.001*	0.401	0.002*
TPA	0.148	0.029*	0.133	0.051	0.297	0.002*	0.300	0.002*	0.655	<0.001*	0.509	<0.001*
FCA	0.025	0.718	−0.005	0.944	−0.095	0.328	−0.097	0.320	−0.240	0.069	−0.251	0.058
JLCA	0.056	0.408	0.005	0.942	−0.076	0.431	−0.095	0.326	−0.144	0.281	−0.116	0.384
Adduction–abduction in the Grood coordinate system, translation (average data)												
FTA	0.130	0.056	0.076	0.263	−0.268	0.005*	−0.189	0.051	−0.275	0.037*	−0.342*	0.009*
MCT	0.068	0.322	−0.032	0.634	0.256	0.007*	0.207	0.032*	0.165	0.215	0.194	0.145
TPA	0.016	0.811	−0.081	0.237	0.234	0.015*	0.193	0.045*	0.234	0.077	0.339	0.009*
FCA	0.119	0.081	0.041	0.550	−0.197	0.041*	−0.113	0.243	−0.057	0.669	−0.065	0.626
JLCA	0.124	0.068	−0.072	0.293	−0.019	0.848	−0.022	0.817	0.014	0.915	−0.089	0.506
Lateral–medial inclination of the femur in the world coordinate system, location (average data)												
FTA	−0.122	0.072	−0.214	0.001*	−0.204	0.034*	−0.369	<0.001*	−0.143	0.286	−0.450	<0.001*
MCT	0.013	0.846	0.058	0.394	0.032	0.743	0.119	0.219	0.120	0.369	0.402	0.002*
TPA	0.048	0.478	0.113	0.096	−0.001	0.991	0.192	0.046*	0.010	0.942	0.393	0.002*
FCA	−0.107	0.115	−0.104	0.128	−0.223	0.021*	−0.202	0.036*	−0.125	0.351	−0.250	0.059
JLCA	0.041	0.553	−0.033	0.624	−0.130	0.180	−0.024	0.805	−0.116	0.384	−0.302	0.021*
Lateral–medial inclination of the femur in the world coordinate system, translation (average data)												
FTA	0.153	0.024*	0.054	0.431	−0.085	0.383	−0.031	0.752	−0.260	0.048*	0.085	0.526
MCT	0.033	0.632	−0.006	0.933	0.190	0.049*	0.209	0.030*	0.066	0.623	−0.294	0.025*
TPA	0.033	0.633	−0.061	0.370	0.148	0.125	0.177	0.067	0.123	0.357	−0.198	0.136
FCA	0.153	0.024*	0.011	0.869	−0.120	0.217	−0.006	0.949	−0.211	0.112	0.071	0.597
JLCA	0.165	0.015	0.050	0.466	0.077	0.430	0.176	0.068	0.023	0.867	−0.002	0.991
Lateral–medial inclination of the tibia in the world coordinate system, location (average data)												
FTA	0.018	0.787	−0.004	0.959	0.147	0.128	0.172	0.075	0.458	<0.001*	0.412	0.001*
MCT	−0.018	0.791	−0.059	0.391	−0.178	0.066	−0.251	0.009*	−0.334	0.010*	−0.412	0.001*
TPA	−0.114	0.094	−0.059	0.389	−0.159	0.100	−0.188	0.052	−0.501	<0.001*	−0.613	<0.001*
FCA	−0.079	0.248	−0.034	0.623	0.035	0.719	0.018	0.850	0.350	0.007*	0.204	0.124
JLCA	−0.017	0.805	−0.048	0.480	0.028	0.775	0.067	0.491	0.063	0.637	0.039	0.774
Lateral –medial inclination of the tibia in the world coordinate system, translation (average data)												
FTA	−0.140	0.039*	−0.007	0.916	0.139	0.151	0.109	0.261	0.206	0.121	0.248	0.061
MCT	0.037	0.585	0.017	0.800	−0.019	0.847	0.019	0.846	−0.204	0.124	−0.169	0.206
TPA	0.015	0.823	0.015	0.830	−0.049	0.617	0.003	0.974	−0.280	0.034*	−0.324	0.013*
FCA	−0.116	0.087	−0.007	0.918	0.240	0.012*	0.204	0.035*	0.078	0.559	0.104	0.436
JLCA	−0.066	0.332	0.097	0.153	−0.044	0.653	0.070	0.471	−0.126	0.347	−0.094	0.485

Abbreviations: CC, correlation coefficient; FCA, femoral condylar angle; FTA, femorotibial angle; JLCA, joint‐line convergence angle; KL, Kellgren–Lawrence classification; MCT, medial compartment of tibia plateau angle; TPA, tibial plateau angle.

* *p* < 0.05.

## RESULTS

Normal motion for KL grades 0 and 1 in the Grood coordinate system (Figures 4, 5, 6) demonstrated flexion, abduction and external rotation as the locations and flexion, adduction and internal rotation as the translations. In the world coordinate system (Figures 7, 8, 9, 10, 11, 12), regarding location, the thigh and shank were inclined posteriorly to anteriorly and laterally, respectively, for both bones in the coronal plane. Regarding rotation, the thigh was internally to externally rotated, whereas the shank was externally rotated. Regarding translation, the thigh and shank were inclined anteriorly and laterally, respectively. Regarding rotation, the thigh was externally rotated, whereas the shank was internally rotated during the loading response phase and subsequently exhibited external rotation.

In OA knees, the location in the Grood coordinate system (Figures 4, 5, 6; Tables 2 and 3) showed flexion (more flexion relative to normal; loading, *p* = 0.006; stance, *p* < 0.001), adduction (abduction in normal; loading, *p* = 0.002; stance, *p* < 0.001) and external rotation (no significant difference from normal), whereas the translation showed flexion (less flexion relative to normal; loading, *p* = 0.013), adduction (more adduction relative to normal; loading and stance, *p* < 0.001) and internal rotation (no significant difference from normal). Regarding the location in the world coordinate system (Figures 7, 8, 9, 10, 11, 12; Tables 2 and 3), the thigh and shank were inclined posteriorly to anteriorly (the thigh was significantly more posterior than normal; loading, *p* = 0.036; stance, *p* < 0.001). In the coronal plane, the lateral inclination of the thigh decreased more than normal (stance, *p* = 0.001), whereas the lateral inclination of the shank increased more than normal (loading and stance, *p* < 0.001). Regarding rotation, the thigh was significantly more externally rotated than normal (loading, *p* = 0.005; stance, *p* = 0.009), whereas the shank was more externally rotated than normal but without a significant difference. Regarding translation in relation to knee OA, the thigh and shank showed anterior inclination to a similar degree as normal. The lateral inclination of the thigh significantly decreased more than normal (loading, *p* < 0.001), whereas that of the shank significantly increased more than normal (loading and stance, *p* < 0.001). Regarding rotation, the thigh (loading, *p* = 0.001; stance, *p* = 0.004) and shank (loading, *p* < 0.001) were significantly more externally rotated than normal.

Regarding the correlation between the joint‐line and motion parameters (Tables 4, 5, 6), the tibial joint inclination correlated with both location and translation in coronal, sagittal and rotational parameters. In the coronal plane, joint‐line inclination exhibited a minimal effect on healthy participants; however, its effect increased with increasing KL grades (Table 6).

## DISCUSSION

The most important findings of the present study were (1) the normal knee motion applied not only the relative motion (Grood coordinate system) but also the individual motion of the thigh and shank (world coordinate system); (2) OA knees demonstrated kinematics different from that of healthy knees, with the main responsibility for the thigh in the sagittal and rotational planes and for the thigh and shank in the coronal plane; (3) the involvement of the joint‐line inclination in kinematics was principally on the tibial side and (4) the effect of joint‐line inclination was less significant in normal knees than in OA knees.

In the sagittal plane, the location of the relative motion (Grood coordinate system) was flexed in OA knees. For each motion of the thigh and shank (world coordinate system), there was no difference in tibial location; however, the thigh was more significantly posteriorly inclined than normal. Therefore, the OA change in the flexed motion of the knee was due to the posterior inclination of the thigh. During walking, the tibial joint‐line inclination attempts to approach parallel to the ground to maintain balance; [12] therefore, the tibial location and translation are assumed to be similar in OA and normal knees. A static standing alignment evaluation showed that the tibial sagittal plane approached more parallel to the ground while standing [14]. Increased tibial flexion contracture and steeper tibial posterior slope have been reported in OA knees [14]. Given this background of impaired knee extension due to bone deformity and soft tissue contracture, even if the tibia may show the same dynamics as normal, the femur cannot fully extend (anterior inclination) from the tibia and the knee as a whole may exhibit relative motion in the flexed position during the stance phase of gait analysis.

Comparing normal and OA knees in the coronal plane, increased adduction in the relative motion (Grood coordinate system) was observed in OA knees. Regarding the individual bones (world coordinate system), the thigh showed a decrease in lateral inclination, whereas the shank showed an increase in lateral inclination in both location and translation in OA knees. The medial tibial joint‐line inclination increases with the progression of knee OA [10]. A static standing alignment study reported that the medial articular surface of the tibia was parallel to the ground (tibial parallel phenomenon) [14]. The tibial lateral inclination in the present study during the gait assumingly indicates the tibial parallel phenomenon during the gait. Regarding the femur, it is abducted by the internal abduction moment of the hip joint [1] because of the need to maintain balance in the one‐leg standing position. Previous alignment research has shown that advanced knee OA causes tibial lateral and femoral medial inclinations, resulting in varus alignment of the entire lower extremity [18]. In summary, while the femur is inclined laterally to maintain balance during walking, it is initially inclined medially due to knee OA; therefore, during walking, the thigh exhibits reduced lateral inclination.

When the rotation was examined in relative motion (Grood coordinate system), no OA change was observed. However, when the location and translation during the loading response phase were examined for each bone (world coordinate system), both showed different rotational kinematics in OA knees compared with normal knees. In particular, external rotation of the thigh was significant. Notably, many studies on the evaluation of static rotational alignment in the supine position have reported no rotational changes in patients with OA knees compared with healthy individuals [19, 22]. However, a standing alignment study reported an internal rotation of the tibia relative to the femur [18], which is a controversial finding. In the present study, there was no difference in rotational kinematics during gait between normal and OA knees when viewed as the relative motion of the two bones. However, compared with normal knees, when OA knees were evaluated individually, each thigh and shank showed external rotational kinematics. In particular, the external rotation of the thigh was greater in patients with knee OA than in individuals with normal knees. This implies that an external rotational force is exerted on the femur during walking due to loading. Therefore, the femur may have an external rotation deformity similar to a torsion deformity because it continues to be subjected to external rotational forces over time. This suggests that the femoral neck anteversion is decreasing. Previous studies have reported that femoral neck anteversion is reduced in patients with knee OA compared with individuals with normal knees [13], which is consistent with the findings of the present study. At first glance, OA knees may be judged to have no abnormal rotation when viewed as a relative motion (Grood coordinate system); however, when evaluated in terms of individual bones (world coordinate system), both bones show external rotational changes, with the thigh, in particular, showing a greater change in rotational motion.

The tibial joint‐line inclination is involved in kinematics. In the present study, the joint‐line inclination correlated not only with coronal plane motion but also with rotational and sagittal plane motion to a lesser extent. This implies that the joint‐ line inclination is involved in 3D motion in planes other than the coronal plane because the bone morphology changes not only in the coronal plane but also in 3D. This finding is consistent with that of previous studies on 3D articular plane inclination, which reported that not only the coronal plane but also the sagittal plane and 3D vectors showed changes in knee OA [11]. The reason for the low involvement of the femoral joint‐line inclination in the present study is that the femoral joint‐line inclination did not significantly differ according to the KL grade. However, a previous longitudinal study showed that the average change in the femoral joint‐line inclination was 1–2° in the same participants over 20–30 years [20]. In a cross‐sectional study such as the present study, it is assumed that the difference is only to the extent that it is absorbed by measurement error, which is why no difference was observed in the KL grade, as in the present study. However, we found a difference in the mean value of the tibial joint‐line inclination according to KL grades, consistent with the findings in previous reports. Therefore, we could indicate its involvement in knee kinematics.

The tibial joint‐line mainly affected the coronal plane kinematics; however, the effect of joint‐line inclination was less in healthy participants. The involvement of joint‐line inclination in kinematics increased with increasing KL grades. The average age of healthy participants at KL grades 0 and 1 was 13 years younger than that of those at higher KL grades in the present study. Given this age difference of >10 years between those at low and advanced OA stages, there were differences in muscle strength and locomotion and the effects of bone morphology were thought to be compensated for by these factors. This finding suggests that if muscle strength and physical ability can be improved, the effects of changes in bone morphology in patients with advanced knee OA can be compensated for to a small extent.

This study has several limitations. First, because this was a field study, a force plate could not be set and kinetic data were not obtained. Second, we measured the joint‐line angle with respect to the anatomical axis using short films. Because there is a strong correlation between the mechanical axis of the full limb and the anatomical axis of the short film [9], the present results are essentially the same as those of studies that used mechanical axes. Future studies are required to assess the full limb using radiography. Third, in the motion capture system, there is a skin movement artifact between the bone and the skin surface to which the reflective markers are applied. This error is considered to be larger in fatty subjects and elderly people with reduced muscle strength. The upper limit of the 95% confidence interval for BMI in this study is 26.6, and extremely obese cases were not included. Generally, the error of the motion capture system with makers is around 3°. The main items of the kinematic parameters between normal and osteoarthritic knees in this study showed a difference of more than 3°. Even if the motion analysis error is taken into account, the results of this study are considered to be clinically meaningful.

## CONCLUSIONS

OA knees demonstrated kinematics different from that of healthy knees, with the main responsibility in the thigh in the sagittal and rotational planes and in both the thigh and shank in the coronal plane. The involvement of joint‐line inclination in kinematics was principally on the tibial side; however, its effect was less significant in normal knees than in OA knees.

## AUTHOR CONTRIBUTIONS

Tomoharu Mochizuki, Hiroshi Koga, Go Omori and Yoshio Koga conceived the study. Tomoharu Mochizuki, Hiroshi Koga, Go Omori and Yoshio Koga designed the study. Tomoharu Mochizuki, Hiroshi Koga, Go Omori, Katsutoshi Nishino, Shigeru Takagi, Hiroshi Koga, Yoshio Koga and Hiroyuki Kawashima collected the data. Fangzhou Chi, Tomoharu Mochizuki and Katsutoshi Nishino analysed the data. Fangzhou Chi and Tomoharu Mochizuki drafted the initial manuscript. All authors gave critical review and advice on the study design and interpretation. All authors contributed to reviewing and revising the manuscript and agreed on the final draft.

## CONFLICT OF INTEREST STATEMENT

The authors declare no conflict of interest.

## ETHICS STATEMENT

The Institutional Review Board of our University (Niigata University) approved this study. All the participants provided informed consent before participating in the survey.
